# Identification of CELSR2 as a novel prognostic biomarker for hepatocellular carcinoma

**DOI:** 10.1186/s12885-020-06813-5

**Published:** 2020-04-15

**Authors:** Mingxing Xu, Shu Zhu, Ruiyun Xu, Nan Lin

**Affiliations:** 1 Department of Hepatobiliary Surgery, The Third Affiliated Hospital of Sun Yat-sen University, No. 600 Tianhe Road, Guangzhou, 510630 Guangdong China; 2 Department of Infectious Diseases, The Third Affiliated Hospital of Sun Yat-sen University, No. 600 Tianhe Road, Guangzhou, 510630 Guangdong China

**Keywords:** CELSR2, Prognosis, Hepatocellular carcinoma, Biomarker

## Abstract

**Background:**

CELSR2 is postulated to be a receptor involved in contact-mediated communication; however, the specific function of this particular member has not been determined in hepatocellular carcinoma (HCC).

**Methods:**

Here, we explored the expression and function of CELSR2 in HCC patients through data mining and examined the results using clinical samples and in vitro experiments.

**Results:**

It was found that CELSR2 mRNA and protein expression levels were significantly higher in cancerous tissue than in normal tissue. The increased mRNA expression of CELSR2 was significantly associated with overall survival (OS) in HCC patients. Moreover, the genetic alteration rate of CELSR2 gene in HCC can reach 8%, and these alterations would deeply influence its neighboring genes, then jointly affecting the occurrence and development of tumor through cell adhesion and numerous common carcinogenic pathways. Our in vitro results indicated that the depletion of CELSR2 inhibited liver cancer cell proliferation and invasion. Univariate and multivariate Cox regression analyses showed that CELSR2 could be viewed as an independent risk factor for HCC patients.

**Conclusions:**

This study demonstrated that data mining could efficiently reveal the roles of CELSR2 in HCC and its potential regulatory networks. The CELSR2 protein level may serve as a novel prognostic biomarker for HCC.

## Background

Hepatocellular carcinoma (HCC) is the fourth most common cause of cancer-related death worldwide [1]. Although diagnosis and treatment for HCC has progressed in recent years, patient prognosis still has much room for improvement. Imaging examinations (e.g., ultrasound imaging (UI), computer tomography (CT) imaging, and magnetic resonance imaging (MRI)) and serum measurements (e.g., alpha-fetoprotein (AFP) and glypican-3 (GPC3)) [2] are the most commonly used methods for early screening and diagnosis of liver cancer. However, the early detection rate of HCC still remains dismal [3]. To achieve early and accurate diagnosis for HCC patients, it is essential to explore new biomarkers.

Cadherin EGF LAG seven-pass G-type receptor 2 (CELSR2) is a member of the flamingo subfamily, part of the cadherin superfamily. The flamingo subfamily consists of nonclassic-type cadherins, a subpopulation that does not interact with catenins [4, 5]. Currently, studies about CELSR2 gene were mainly focused on nervous system disease, coronary artery disease and process of serum cholesterol metabolism [5–8]. Additionally, functions of CELSR2 were also reported in some tumors. In breast cancer cells, CELSR2, together with inhibitor of growth 4 (ING4) displayed increased cytoplasmic staining compared to benign epithelium cells, suggesting a possible role of both genes in the pathogenesis of human mammary neoplasia [9]. In Kakehashi A et al.*’*s study, CELSR2 was validated to participate in promoting mammary and endometrial carcinogenesis and altering the molecular tumor environment [10]. In prostate cancer, methylation of CELSR2 has been shown to play an important role in carcinogenesis and tumor progression [11]. Given the increasing importance of CELSR2 in tumors, and currently there is rare research about the diagnostic and prognostic values of CELSR2 in HCC; hence, related study is urgently needed.

In this study, we systematically explored the function of CELSR2 in HCC using bioinformatics data mining and clinical samples. Our results showed that both the gene and protein levels of CELSR2 were differentially overexpressed in cancerous tissues comparing to adjacent normal tissues. Data from The Cancer Genome Atlas (TCGA), the GeneExpression Omnibus (GEO) and Genotype-Tissue Expression (GTEx) project indicated that the coexpression networks in cancerous tissue, adjacent liver tissue and normal liver tissue were different. Moreover, CELSR2 was a prognostic risk factor, and low expression was favorable in HCC. In addition, genetic alteration of CELSR2 and its neighboring genes were analyzed in HCC to reveal that these genes could jointly affect the occurrence and development of tumors through common carcinogenic pathways.

## Methods

### Human protein atlas

The Human Protein Atlas (HPA) (https://www.proteinatlas.org) is a website tool that contains gene expression data of nearly 20 highly common kinds of cancers, and each tumor type includes 12 individual tumors [12]. The mRNA and protein levels of CELSR2 expression in both normal tissues and cancerous tissues were evaluated using this tool. In addition, the subcellular localization of CELSR2 was also validated using the HPA database.

### HCCDB

The HCCDB database (http://lifeome.net/database/hccdb) is a free one-stop online resource for exploring HCC gene expression with a user-friendly interface. It includes 15 datasets that cover approximately 4000 clinical samples [13]. Users can analyze the consistently differentially expressed genes across multiple datasets to establish a global differential gene expression landscape of HCC. Gene expression in various liver tissues and coexpression networks in cancerous tissue, adjacent liver tissue and normal liver tissue all can be analyzed using this tool.

### UALCAN

UALCAN (http://ualcan.path.uab.edu) is an online tool whose resource mainly comes from the level 3 RNA-seq and clinical data of 31 cancer types from the TCGA database. This tool is commonly used when analyzing gene expression profiles and relationships between mRNA expression and clinical characteristics [14].

### Kaplan-Meier plotter

The Kaplan-Meier plotter (http://kmplot.com/analysis/) is a well-known and widely used online survival analysis tool. In this study, the prognostic value of CELSR2 overexpression in HCC samples was analyzed using this tool.

### cBioPortal and g:profiler

The cBio Cancer Genomics Portal (http://cbioportal.org), as an online analysis tool, is mainly used for the exploration of multidimensional cancer genomics data sets whose resource comes from more than 5000 tumor samples of 20 cancer studies [15]. In this study, Gene Ontology (GO) and KEGG pathway enrichment analyses of CELSR2 and its neighboring genes were performed with the g:Profiler (http://biit.cs.ut.ee/gprofiler/) online tool [16]. GO annotation has three parts: cellular component (CC), biological process (BP), and molecular function (MF).

### LinkedOmics

The LinkedOmics database (http://www.linkedomics.org/login.php) is an open access online biometrics platform whose resource comes from 11,158 patients from the TCGA [17]. In this study, we analyzed genes differentially expressed in correlation with CELSR2 in the TCGA HCC cohort (*n* = 371), committed to finding and assessing the correlation between genes by Pearson’s correlation coefficient. Similarly, the Web-based GEne SeT AnaLysis Toolkit (WebGestalt) [18] was then used to perform GO (CC, BP and MF), KEGG pathway, kinase-target enrichment, miRNA-target enrichment and transcription factor-target enrichment analyses of these related genes.

### Patient samples and cell lines

Seventy-four pairs of fresh human HCC samples and corresponding normal non-cancerous tissues were obtained during surgery at the Department of Hepatobiliary Surgery from the Third Affiliated Hospital of Sun Yat-Sen University (Guangzhou, China). All samples were collected with patients’ informed consent. The basic clinical features of all the participants are summarized in Additional file 1: Table S1.

Human hepatoma cell lines (HepG2, Hep3B, and Huh7) and immortalized liver cell (LO2) were used for in vitro experiments, which were purchased from Shanghai Institutes for Biological Sciences, Chinese Academy of Sciences (Shanghai, China). Cells were maintained in Dulbecco’s Modified Eagle Medium (DMEM; Gibco, Carlsbad, USA) supplemented with 10% fetal bovine serum (FBS) and 10 μ/ml penicillin G/streptomycin at 37 °C in a humidified atmosphere containing 5% CO2.

### Immunohistochemistry analysis

Formalin-fixed HCC tissue samples were used to perform this assay according to the manufacturer’s instruction. After incubation with anti-CELSR2 primary antibody (Cell Signaling Technology, USA) and anti-glyceraldehyde phosphate dehydrogenase (GAPDH) primary antibody (Abcam, USA), the sections were then incubated with the corresponding secondary antibody (Abcam) for 1 h at room temperature. Phosphate buffered saline (PBS) was used as negative controls (NC). Semiquantitative analysis of the obtained images was performed using the Image-Pro Plus 6.0 software. A score, calculated by multiplying the staining intensity by the area of positively-stained cells, was assigned for each image.

### Western blot and real-time quantitative polymerase chain reaction

Total protein was extracted from cultured cells using RIPA buffer (Beyotime, China) supplemented with protease inhibitor cocktail (Roche, Switzerland). Proteins were separated by 8% or 10% SDS-PAGE and then transferred to nitrocellulose membranes (0.2 μm and 0.45 μm). After incubation with anti-CELSR2 antibody, HRP-conjugated secondary antibody (Abcam) was used at room temperature for 1 h. The protein expression was detected using an enhanced chemiluminescence kit (ECL; Pierce, USA) according to the manufacturer’s instructions. GAPDH was used as a loading control.

Total RNA was extracted from cultured cell lines using TRIzol reagent (Invitrogen, USA). Total RNA (1 μg) was reverse transcribed into cDNA by the GoScript™ Reverse Transcription System (Promega, USA). SYBR Green (Promega) in Roche LightCycler 96 (Roche Applied Science, Germany) was used to perform the real-time quantitative polymerase chain reaction (qPCR). The primer sequences were as follows: for CELSR2, forward 5′-ACAGCAAAAGAGAGTAGTGGCAAC-3′, reverse 5′-CTTAAGGATGCCTTTGTGAGGC-3′; for GAPDH, forward 5′-GGAGCGAGATCCCTCCAAAAT-3n ′, reverse 5′-GGCTGTTGTCATACTTCTCATGG-3′. Expressions of target gene were normalized to GAPDH and calculated by the 2^–ΔΔCT^ method.

### RNA interference

HepG2 and Hep3B hepatoma cell lines in 6-well plates were transfected with CELSR2 small inference RNA (siRNA) and NC by Lipofectamine 2000 (Invitrogen), which were designed and synthesized by RiboBio company (Guangzhou, China) at a final concentration of 15 nM. Forty-eight hours later, the inhibition efficiency was identified by western blot analysis (Additional file 2: Figure S1, Additional file 3: Figure S2).

### Cell counting kit-8 and invasion assays

Cell proliferation capacity was evaluated using cell counting kit-8 (Biotechwell, China) according to the manufacturer’s instructions. Briefly, treated HepG2 and Hep3B hepatoma cell lines were seeded into 96-well plate at a density of 1 × 10^3^ cells per well and placed at 37 °C in humidity incubator. Then, 10-μl kit solution was added into each well for 1 h at 37 °C for 4 days. The absorbance at 490 nm of each well was recorded with a plate reader.

For migration assay, costar transwell plates with 8-μm pore size (Corning, USA) were used. Cells (1 × 10^5^) in 100-μl DMEM medium without FBS were seeded in triplicate into the upper chamber. To the lower chamber, 600-μl medium containing 10% FBS were added. After 24 h of incubation, the plate inserts were removed and washed with PBS buffer to remove the unattached cells. Residual cells on the upper side were then scraped with a cotton swab. Cells on the lower side of the insert were fixed in 4% formalin for 15 min, washed with PBS twice, and stained with 0.1% crystal violet for 5 min. Finally, five randomly fields were selected for each insert and then were photographed and counted under a light microscope (Leica, Germany).

### Statistics and analysis

Univariate and multivariate analyses were performed using the Cox proportional hazards model. All analyses were performed using SPSS version 22.0 (IBM, United States). A 2-tailed *P* value less than 0.05 was considered statistically significant.

## Results

### mRNA and protein expression profiles of CELSR2 in the HPA

**Fig. 1:**
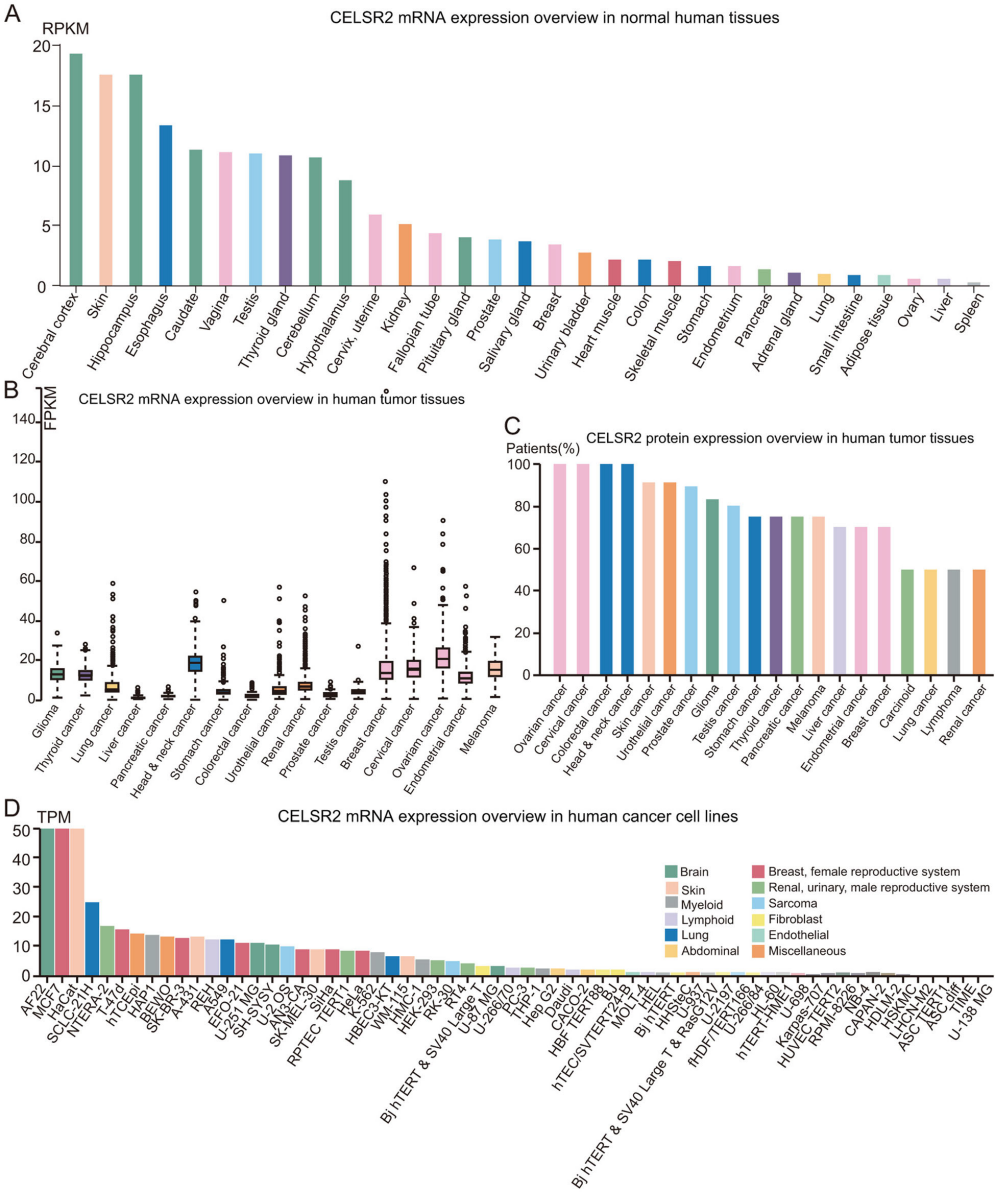
Gene and protein expression profiles of CELSR2 in human normal and tumor samples. **a** CELSR2 mRNA expression data from the GTEx project in the Human Protein Atlas. **b** CELSR2 gene expression in common human tumor tissues in the Human Protein Atlas. **c** CELSR2 protein expression overview in human tumor tissues in the Human Protein Atlas. **d** CELSR2 mRNA expression overview in human cancer cell lines in the Human Protein Atlas

By examining the CELSR2 expression profile in the HPA, we found that the mRNA expression of CELSR2 in normal liver tissues was relatively low compared with that in other human tissues (Fig. 1a). Similarly, as shown in Fig. 1b, the mRNA level of CELSR2 in liver cancer samples was the lowest among all other cancer types. However, at both the mRNA and protein levels, the expression of CELSR2 was significantly upregulated in liver cancer tissues and liver cancer cell line (e.g., Hep G2) compared with other organ tissues and cancer cell lines (Fig. 1c, d). Hence, the CELSR2 protein level, rather than the gene expression level, may be a more sensitive biomarker for HCC diagnosis. Beisdes, in terms of subcellular localization, it can be concluded that the protein localization of CELSR2 in cell lines (e.g., A-431 and U-251 MG) was almost enriched in the cytosol (Additional file 4: Figure S3).

### Expression profile and coexpression network of CELSR2 in the HCCDB

**Fig. 2:**
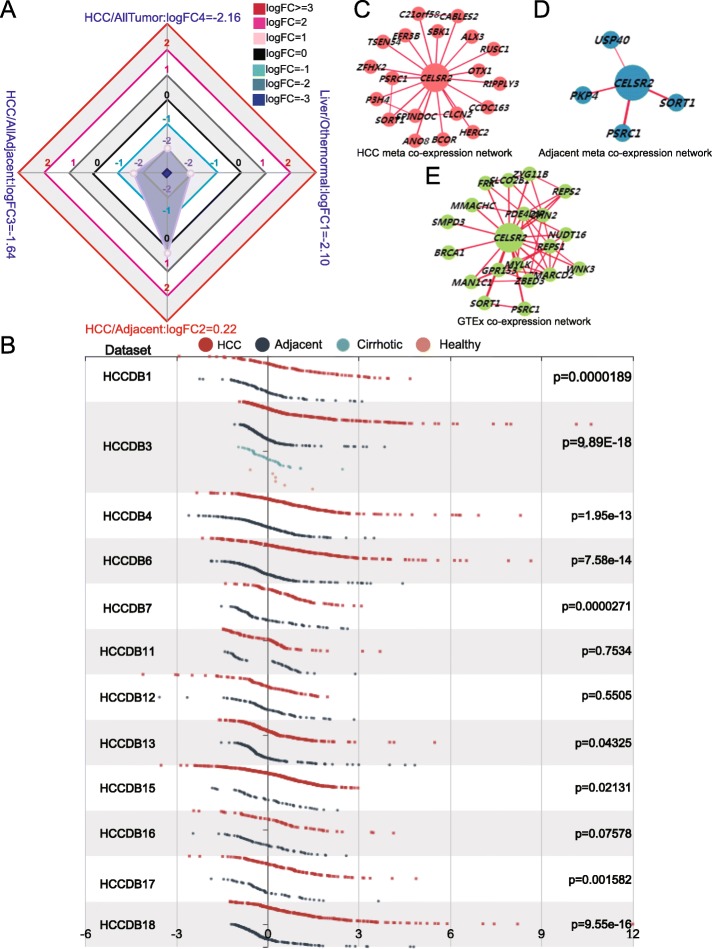
Gene expression profiles of CELSR2 in the HCCDB database. **a** Radar map of CELSR2 overall expression among different types of tissues. **b** The differential expression of CELSR2 in different liver cancer datasets (HCCDB1, HCCDB3, HCCDB4, HCCDB6, HCCDB7, HCCDB13, HCCDB15, HCCDB17 and HCCDB18) suggests that CELSR2 expression is much higher in HCC tissues than in adjacent liver tissues. The coexpression networks of CELSR2 in HCC tissues (**c**), adjacent liver tissues (**d**) and normal tissues from the GTEx project (**e**) showed that different liver tissues expressed different coexpression networks

The radar chart shows the overall expression of CELSR2 among different types of tissues. As shown in Fig. 2a, the gene expression of CELSR2 in liver tissue was lower than that in other normal tissues (liver/other normal: logFC = − 2.10), and CELSR2 expression in HCC was lower than that in other tumor tissues (HCC/all tumor: logFC = − 2.16), which were consistent with results in the HPA (Fig. 1a, b). However, when comparing HCC tissues with adjacent tissues, the gene expression of CELSR2 in HCC tissues was higher than that in adjacent liver tissues (HCC/adjacent: logFC = 0.22). Thenafter, differential expression levels of CELSR2 were detected in 12 different HCC datasets, and the results showed that in most datasets (9/12), such as HCCDB1, HCCDB3, HCCDB4, HCCDB6, HCCDB7, HCCDB13, HCCDB15, HCCDB17 and HCCDB18, the gene expression of CELSR2 in HCC was much higher than that in adjacent liver tissues (Fig. 2b). Finally, we also analyzed the coexpression networks, and the results showed that the coexpression networks of CELSR2 in HCC tissue, adjacent tissue and normal liver tissue were totally different (Fig. 2c-e).

### Association of the CELSR2 mRNA level with clinicopathological parameters in HCC patients

**Fig. 3:**
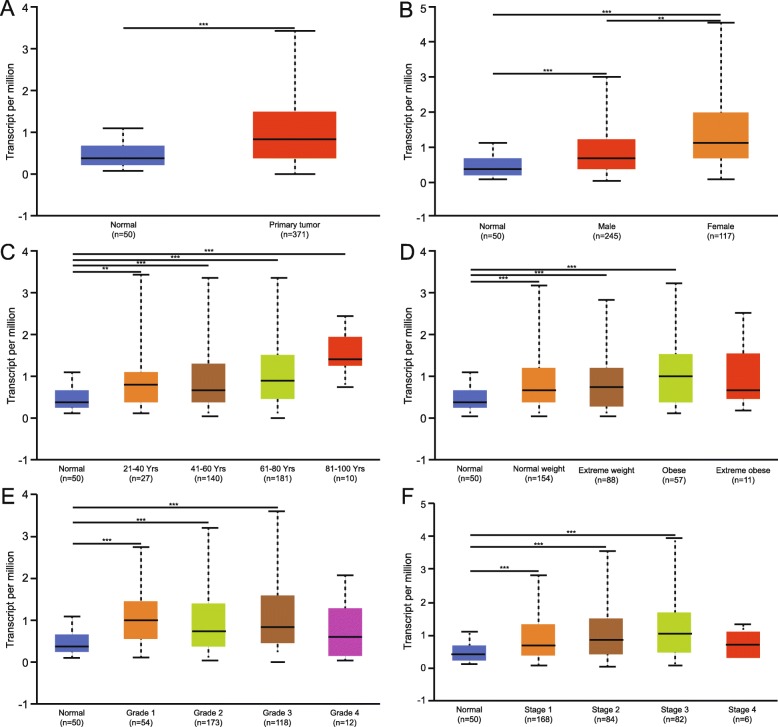
The mRNA expression of CELSR2 in the UALCAN database. **a** The mRNA expression level of CELSR2 was significantly higher in cancer tissues than in normal tissues. **b** The expression level of CELSR2 in female patients was higher than that in healthy people or in male patients. **c** Patients (age > 21 years old) commonly had higher gene expression than young healthy people. The expression level of CELSR2 was positively correlated with patient weight (**d**), tumor stage (**e**) and tumor grade (**f**) in HCC patients. *** represents *p* < 0.001

As shown above, CELSR2 mRNA and protein were overexpressed in cancerous tissue compared with adjacent tissue. We then analyzed the associations between the mRNA expression of CELSR2 and clinicopathological parameters in HCC patients with UALCAN. As shown in Fig. 3a, the mRNA expression of CELSR2 in normal tissue was lower than that in cancerous tissue. The expression level in female HCC patients was higher than that in male patients and healthy people (Fig. 3b). In addition, patients (age > 21 years old) commonly had higher expression level than healthy people (Fig. 3c). Besides, along with weight gain, the expression of CELSR2 showed an increasing trend in HCC (Fig. 3d). Finally, we analyzed the relationship between mRNA expression and tumor grade or stage and found that the mRNA expression level of CELSR2 was positively correlated with tumor stage in HCC patients, which indicated high CELSR2 expression was probably associated with poor clinical characteristics (Fig. 3e, f).

### Prognostic value of the mRNA expression of CELSR2 in HCC patients

**Fig. 4:**
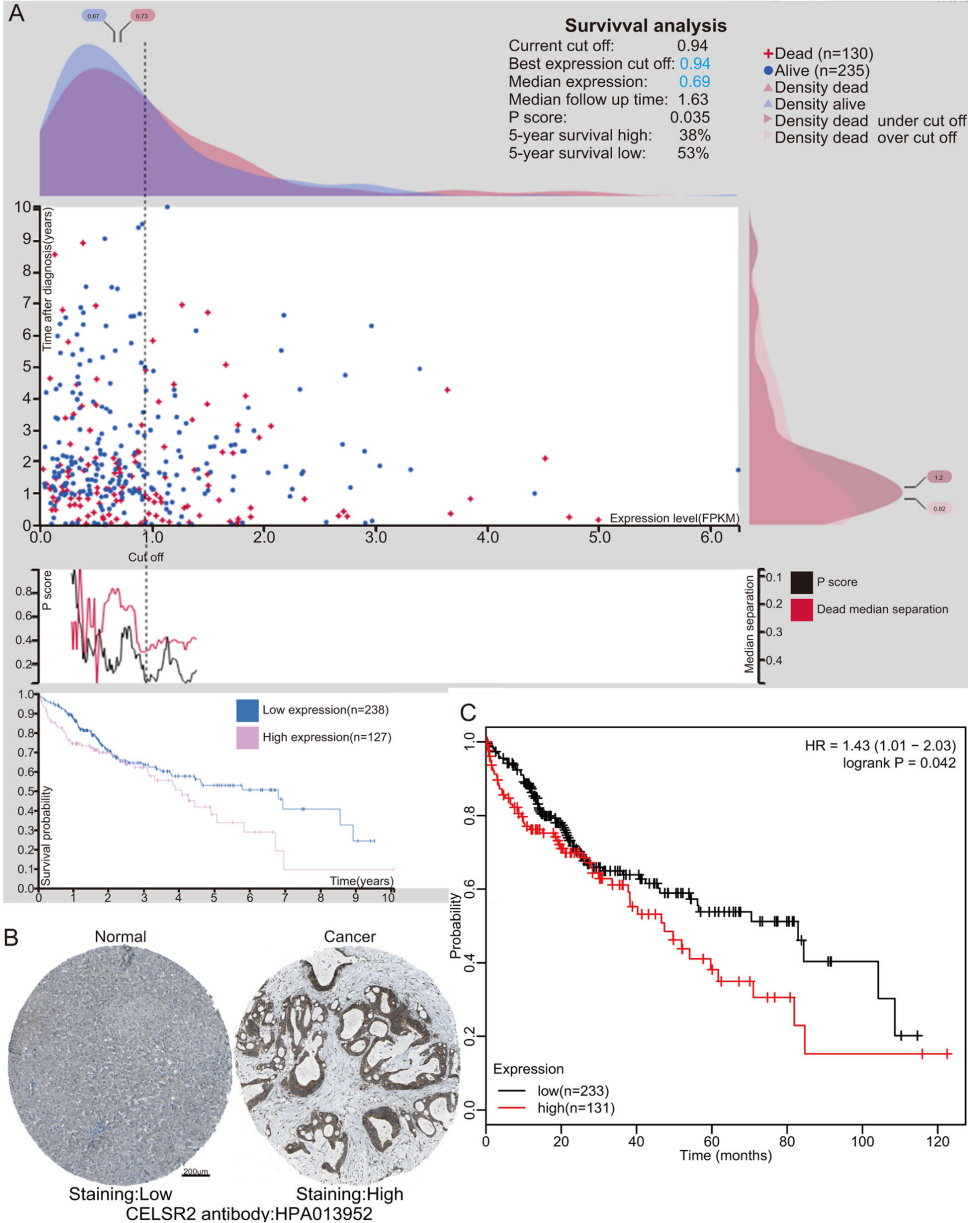
Gene expression of CELSR2 and its prognostic value in HCC patients. **a** Prognostic value of the CELSR2 level in HCC patients from the Human Protein Atlas. **b** Representative immunohistochemistry (IHC) images from the Human Protein Atlas with the CELSR2 antibody: HPA013952, cancerous tissue had higher staining than that in normal liver tissue. **c** High CELSR2 mRNA expression was significantly related to poor OS in HCC patients from the Kaplan-Meier plotter

As shown in Fig. 4b, the protein level of CELSR2 was much higher in HCC samples than in normal tissues from HPA database. We next explored the relationship between the expression level of CELSR2 and patient survival rate using the Kaplan-Meier plotter tool and found that high CELSR2 expression was significantly associated with a poor prognosis in HCC patients (Fig. 4c, Hazard Ratio (HR) = 1.43, 95% confidence interval (CI): 1.01–2.03, *p* = 0.042). To further validate this conclusion, we conducted a survival analysis in the HPA, which indicated a consistent conclusion that high CELSR2 expression is unfavorable in HCC patients (Fig. 4a).

### Genomic alterations and the biological interaction network of CELSR2 in HCC

**Fig. 5:**
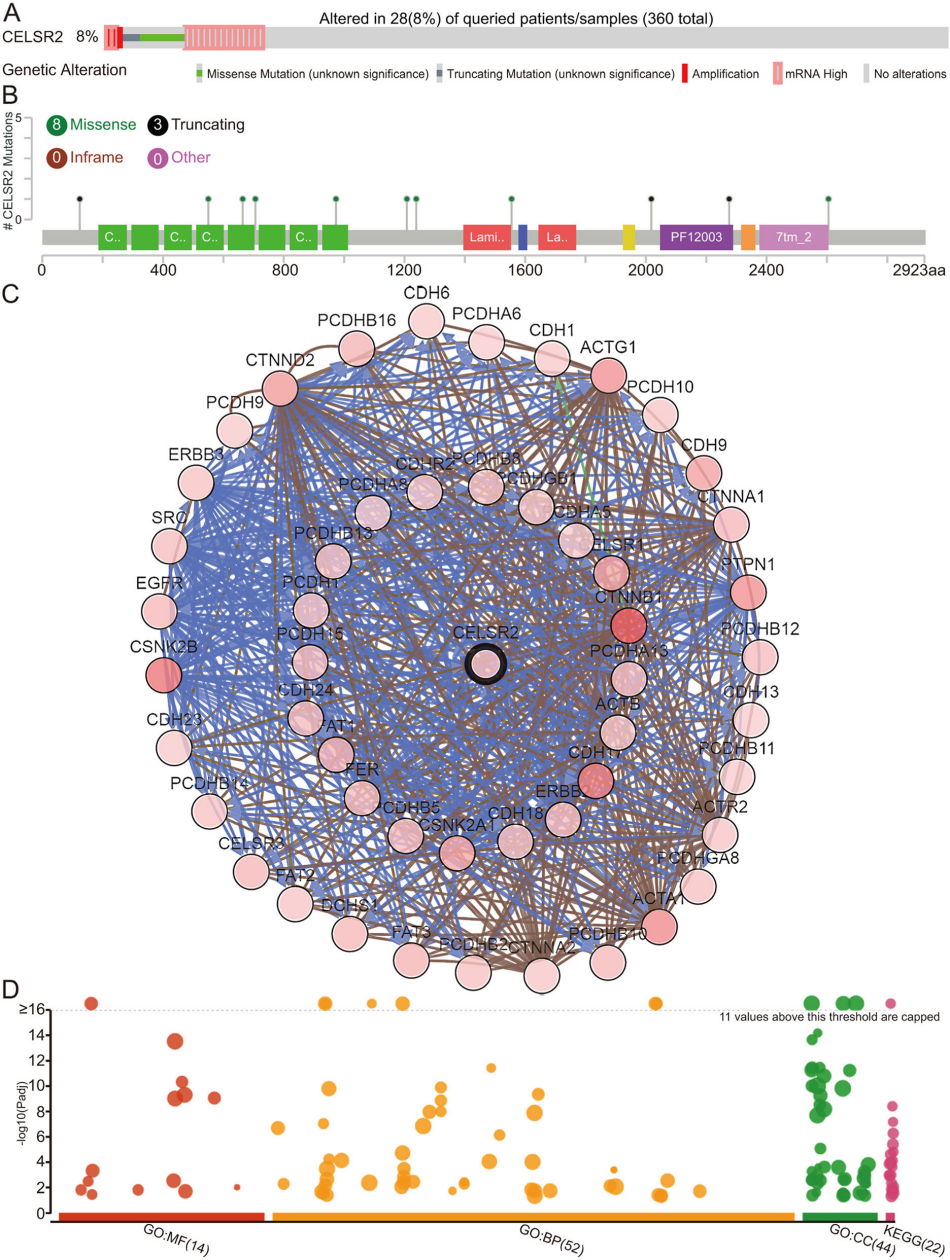
Genetic mutations of CELSR2 and its associations with OS in HCC patients (cBioPortal). **a** Oncoprint of CELSR2 alterations in HCC. The overview of genomic alterations showed that the alteration rate of CELSR2 was 8%. **b** Mutation types of CELSR2 gene in HCC patients. **c** Network view of CELSR2 and its altered neighboring genes in HCC. **d** GO functional enrichment and KEGG pathway analyses of CELSR2 and its frequently altered neighboring genes

Next, TCGA sequencing data from the cBioPortal database were used to explore the genetic alterations of CELSR2 and its association with neighboring genes in HCC patients. As shown in Fig. 5a, CELSR2 was altered in 28 of 360 (8%) patients. Among these alterations, 14 patients had high CELSR2 mRNA expression (4.01%), 11 patient had genetic mutations (3.15%), 2 patients had multiple alterations (0.57%), and 1 patients had amplification (0.29%). Furthermore, mutation diagram showed the corresponding mutation types of CELSR2 in HCC patients from TCGA (Fig. 5b), it could be found that missense mutation was the most common type (8/11), and 3 patients had truncating mutations (Additional file 5: Table S2).

We then analyzed the neighboring genes that were significantly associated with CELSR2 mutations using the Network tool in cBioPortal, and Fig. 5c shows the constructed network. CTNNB1 (27.8%), CSNK2B (20.6%) and CDH17 (18.3%) were the top 3 mutant genes (Additional file 6: Table S3). Next, the functions of CELSR2 and 50 frequently altered neighboring genes were enriched with g:Profiler tool (http://biit.cs.ut.ee/gprofiler/). As shown in Fig. 5d and Additional file 7: Table S4, cellular components, including cell periphery, plasma membrane, catenin complex, cell to cell adherens junction and adherens junction were the 5 most common subcellular localization associated with CELSR2 alterations, which was consistent with the property that CELSR2 as a membrane protein (Additional file 4: Figure S3). In addition, these genetic alterations were primarily involved in homophilic cell adhesion, cell to cell adhesion, cell adhesion, biological adhesion and cell junction assembly biological processes. Moreover, CELSR2 mutations also prominently affected molecular functions, such as the ion binding, cadherin binding, cell adhesion molecule binding, cation binding and cytoskeletal protein binding, which were consistent with the characteristic as a receptor involved in contact-mediated communication. Finally, we conducted KEGG analysis, and the results indicated that pathway associated with the adherens junction, bacterial invasion of epithelial cells, common solid tumors and Hippo signaling was significantly associated with CELSR2 alterations in HCC.

### GO and KEGG pathway analyses of the coexpressed genes correlated with CELSR2 in HCC

**Fig. 6:**
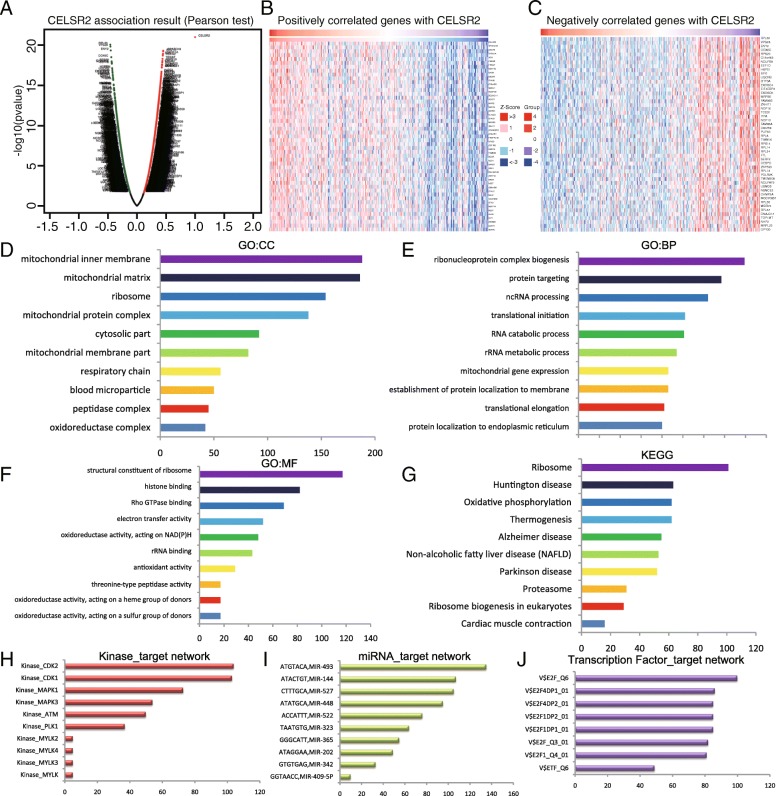
Genes differentially expressed in correlation with CELSR2 in HCC (LinkedOmics). **a** Volcano plot showing genes correlated with CELSR2 through Pearson’s test analysis. **b, c** Heat maps showing the top 50 genes positively and negatively correlated with CELSR2in HCC; red (positive), green (negative). The significantly enriched GO annotations (**d**) cellular components, (**e**) biological processes and (**f**) molecular functions), KEGG pathways (**g**), kinase (**h**), miRNA (**i**) and transcription factor (**j**) targets of CELSR2 coexpressed genes in HCC were analyzed using GSEA

The LinkedOmics database, containing mRNA sequencing data of 371 HCC patients in the TCGA, was used to analyze the coexpressed genes correlated with CELSR2 in HCC. As shown in Fig. 6a, the volcano plot indicated genes with significant positive and negative correlations with CELSR2 (false discovery rate [FDR] < 0.01). The heat map showed that the 50 significant gene sets were positively and negatively correlated with CELSR2 (Fig. 6b, c, Additional file 8: Table S5). We then used gene set enrichment analysis (GSEA) to conduct GO term and KEGG analyses. The results showed that significant genes differentially expressed in correlation with CELSR2 were located mainly in the mitochondria, ribosome, cytosolic part and respiratory chain (Fig. 6d). These genes are mainly involved in ribonucleoprotein complex biogenesis, protein targeting, ncRNA processing, translational initiation and RNA catabolic processes (Fig. 6e). Moreover, they play important roles in structural constituent of ribosome, histone binding, Rho GTPase binding, electron transfer activity and oxidoreductase activity (Fig. 6f). Finally, KEGG pathway analysis showed that these genes are mainly enriched in ribosome, oxidative phosphorylation, non-alcoholic fatty liver disease, proteasome and ribosome biogenesis pathways (Fig. 6g).

### CELSR2 networks of kinase, miRNA or transcription factor targets in HCC

To further investigate the targets of CELSR2 in HCC, the kinase, miRNA and transcription factor target networks of positively correlated gene sets were analyzed by GSEA. As shown in Fig. 6h, the top 5 most significant kinase target networks were cyclin-dependent kinase 2 (CDK2), CDK1, mitogen-activated protein kinase 1 (MAPK1), MAPK3 and ATM (ataxia-telangiectasia, mutated) kinase. The miRNA target network showed that (ATGTACA) MIR-493, (ATACTGT) MIR-144, (CTTTGCA) MIR-527, (ATATGCA) MIR-448 and (ACCATTT) MIR-522 were the most significant gene sets (Fig. 6i). In addition, the transcription factor target network was related mainly to the E2F transcription factor family, including E2F_Q6, E2F4DP1_01, E2F4DP2_01, E2F1DP2_01 and E2F1DP1_01 and ETF (TEA domain family member 2)_Q6 (Fig. 6j).

### Validation of CELSR2’s roles in HCC

**Fig. 7:**
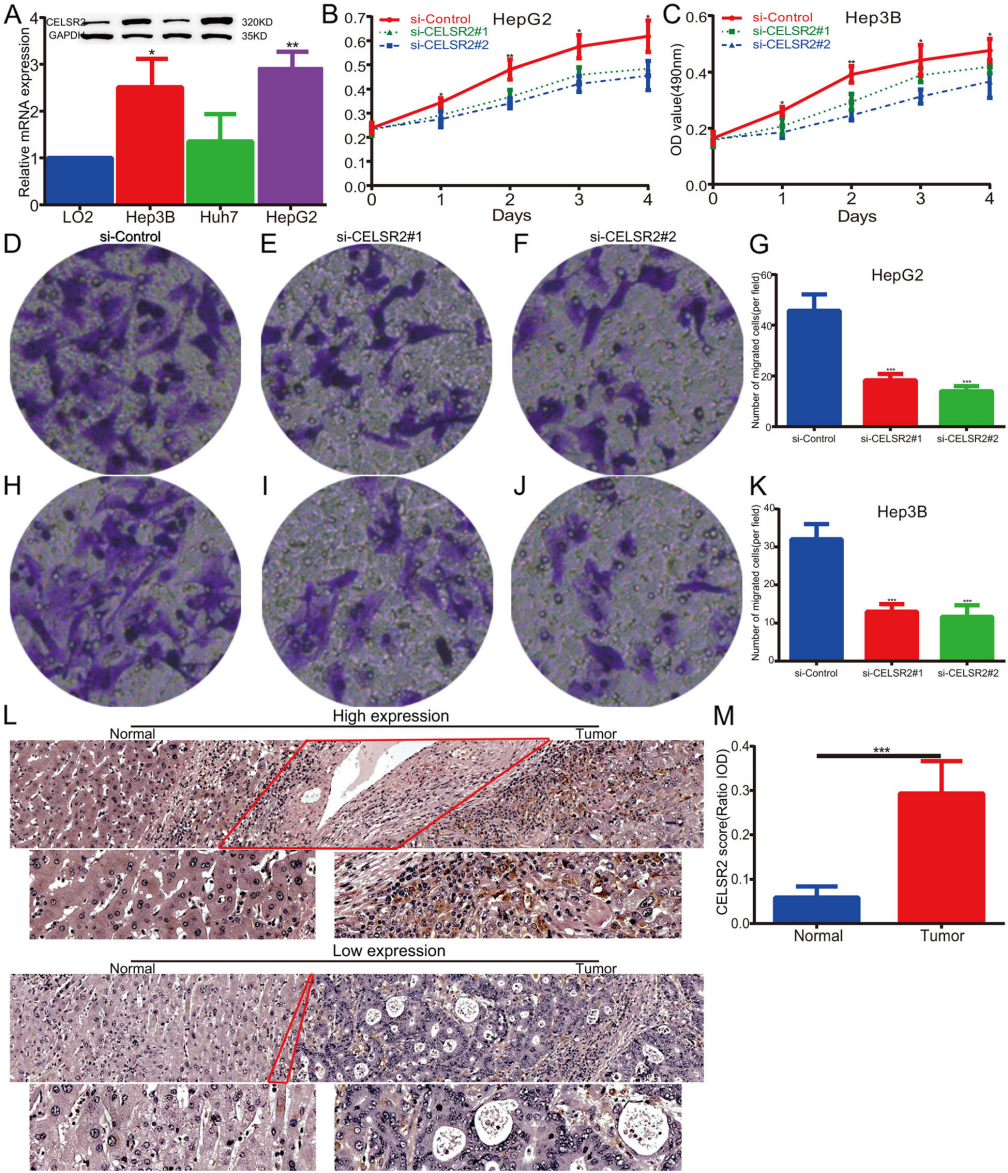
Expression and biological function of CELSR2 in HCC. **a** The mRNA and protein levels of CELSR2 from cultured cells by qPCR and western blot assays. The expression of CELSR2 protein was normalized to GAPDH. **b, c** CELSR2 knockdown prohibited the proliferation rate of hepatoma cells. **d-k** CELSR2 knockdown reduced cell invasion in both HepG2 and Hep3B cells. **l** Representative cases of high and low CELSR2 expression by immunohistochemistry staining. **m** Semi-quantitative analysis of CELSR2 protein expression between cancerous specimens and non-cancerous parts. * represents *p* < 0.05, *** represents *p* < 0.01, *** represents *p* < 0.001

To confirm the conclusions above, we measured the expression level of CELSR2 in cultured hepatoma cell lines and HCC specimens. The results showed that CELSR2 was upregulated in hepatoma cell lines, especially in HepG2 and Hepa3B compared with LO2 (Fig. 7a, *p* < 0.01, Additional file 3: Figure S2). Then, we constructed HCC cell models of CELSR2 knockdown with two distinct siRNA duplexes to investigate the biological function of CELSR2 in HCC. As shown in Fig. 7b-k, CELSR2 knockdown was found to significantly inhibit HCC cell proliferation and invasion abilities compared with the control group. Finally, protein level was detected in HCC specimens and matched non-tumor specimens. It can be found that CELSR2 was upregulated in HCC specimens compared with matched normal tissues (Fig. 7l, m). Meanwhile, univariate and multivariate analyses were used to analyze the prognostic values of CELSR2 in HCC. On univariate analysis, AFP level, liver cirrhosis and CELSR2 expression were identified as significant factors of recurrence-free survival (RFS). Multivariate analysis revealed that CELSR2 level (HR 21.693; 95% CI, 7.790–60.409; *p* < 0.01), together with patient age (HR 1.038; 95% CI, 1.004–1.074; *p* = 0.03) and liver cirrhosis (HR 2.670; 95% CI, 1.019–6.996; *p* = 0.046) were significantly associated with RFS (Table 1). Similarly, based on multivariate analysis, patient age, tumor size, liver cirrhosis and CELSR2 expression were significantly related to overall survival in HCC patients (Table 2).

**Table 1:** Prognostic factors associated with recurrence-free survival

	Univariate analysis			Multivariate analysis		
	*p* value	HR	95% CI	*p* value	HR	95% CI
Gender	0.471					
Age	0.736			0.030	1.038	1.004–1.074
Tumor size	0.527					
Tumor number	0.446					
Vascular invasion	0.659					
PLT	0.357					
ALT	0.542					
AFP	0.050	1.000	1.000–1.000			
HbsAg	0.838					
HBV-DNA	0.718					
Operative time	0.203					
Liver cirrhosis	0.010	2.922	1.287–6.634	0.046	2.670	1.019–6.996
Tumor differentiation	0.387					
MVI	0.859					
CELSR2	< 0.01	10.924	5.159–23.131	< 0.01	21.693	7.790–60.409

**Table 2:** Prognostic factors associated with overall survival

	Univariate analysis			Multivariate analysis		
	*p* value	HR	95% CI	*p* value	HR	95% CI
Gender	0.416					
Age	0.116			0.039	1.075	1.004–1.152
Tumor size	0.458			0.013	1.398	1.074–1.821
Tumor number	0.717					
Vascular invasion	0.375					
PLT	0.910					
ALT	0.612					
AFP	0.004	1.000	1.000–1.000			
HbsAg	0.389					
HBV-DNA	0.319					
Operative time	0.980					
Liver cirrhosis	0.036	3.667	1.089–12.348	0.003	18.624	2.622–132.303
Tumor differentiation	0.465					
MVI	0.456					
CELSR2	< 0.01	70.672	9.450–528.498	< 0.01	1173.999	34.682–39,740.677

## Discussion

Although comprehensive measures have shown their efficacy in preventing HCC and in curbing overall mortality from the disease, incidence and cancer-specific mortality still are at high levels. Moreover most HCC patients are in advanced stage and the prognosis is not satisfactory [19–21]. There are many treatments for liver cancer, and the radical methods include hepatic resection [22, 23], radiofrequency ablation (RFA) [24, 25], and liver transplantation [26, 27]. Early diagnosis and early treatment have always been the focus for liver cancer, but research results over the years are not impressive. Hence, exploring novel diagnostic and prognostic biomarkers has become a hot spot in this field.

CELSR2 is the mammalian orthologue of flamingo, the planar cell polarity protein that belongs to a unique cadherin subfamily. Structurally, CELSR2 is a nonclassic member of the cadherin family containing seven transmembrane and nine cadherin domains as well as seven EGF-like and two laminin AG-type repeats. Commonly, CELSR2 is considered to play an important role in the development of the nervous system, specifically in dendrites and axon outgrowth in the mammalian nervous system [7, 28]. In solid tumors, the functions of CELSR2 are obtaining more and more attention from researchers. In breast cancer, CELSR2 was down-regulated in HER2-positive breast carcinoma [29]. In the endometrial adenocarcinomas, induction of CELSR2 could be found in the process of carcinogenesis [10]. In addition, methylation of CELSR2 has been shown to play an important role in carcinogenesis and tumor progression in prostate cancer [11]. However, to the best of our knowledge, there is almost no evidence showing the roles of CELSR2 in HCC. Considering the roles of CELSR2 in other tumors, we hypothesized this study on the function and mechanism of CELSR2 in HCC, which was urgently needed to better elucidate the occurrence and progression of this tumor.

We first detected the expression of CELSR2 at both the mRNA and protein levels in normal human organs and common cancers using the HPA database. Intriguingly, although CELSR2 mRNA expression was relatively low in both normal and cancerous tissues from the liver compared with that from other organs, its protein level was significantly upregulated in cancerous liver tissues. These seemingly paradoxical results may reflect that the hepatic translational efficiency of CELSR2 after transcription is much higher than that of other organs. Of course, this conjecture and possible internal regulatory mechanisms need to be confirmed by a large number of basic studies in the future. Then, we focused on the difference in CELSR2 expression levels between normal liver tissue and HCC tissue. A number of different data platforms have shown that CELSR2 expression in liver cancer tissues was significantly higher than that in normal liver tissues, both in terms of the mRNA and protein levels, and these results indicated to some extent that this gene perhaps would play a carcinogenic role in the occurrence of liver cancer.

To further explore the prognostic value of CELSR2 in HCC, we subsequently conducted Kaplan-Meier analysis in the TCGA HCC cohort and found that high CELSR2 expression correlated well with a poor prognosis. Moreover, the value of CELSR2 in HCC was also validated using hepatoma cell lines and clinical samples, and the results were consistent with the conclusions analyzed by public database. This is the first report to decipher the associations of CELSR2 with patient prognosis in HCC patients. Considering genetic alterations or dysregulated amplification is believed to play an important role in the development of many tumors [30–32], we thus explored the intrinsic carcinogenesis mechanism of CELSR2 in liver cancer by detecting the genetic alterations of CELSR2. In this study, an 8% genetic alteration rate of CELSR2 gene was observed in HCC, and the alteration of this gene was significantly associated with 50 neighboring genes. To better explore the roles of genetic alterations of these genes in HCC, we performed functional enrichment analysis. The functional networks of neighboring genes close to CELSR2 are generally involved in cell adhesion and intercellular interaction processes, and mainly functioned through adhesion-related signaling pathways, which was consistent with previous studies [33–35]; meanwhile, they also worked through carcinogenic signaling pathways e.g., the classic Hippo signaling pathway in HCC [36–38]. However, large amount of verification experiments were still needed in the future.

Important networks of target kinases, miRNAs and transcription factors can be explored when conducting an enrichment analysis of target gene sets using GSEA. In the present study, the functional network of CELSR2 transcription is involved in transcription factors ETF and E2F, which are important factors in hepatocyte proliferation and chemotherapeutic efficacy in HCC patients [39, 40]. These findings showed the role of CELSR2 in the cell cycle and cell function regulation, giving us more reason to believe that the occurrence of cancer, including HCC, may be affected by this gene.

## Conclusion

Taken together, these results provide multilevel evidence for the importance of CELSR2 in hepatocarcinogenesis and its potential as a marker in HCC. This study used online tools based on the most popular bioinformatics theories to perform target gene analyses on tumor data from public databases. Compared with traditional chip screening, this method has the advantages of a large sample size, low costs, and simplicity. However, due to limited HCC cohorts, we could not extend the external verification of this prognostic model. Future work will focus on utilizing clinical parameters along with the biomarker to improve its performance in HCC. In addition, the exact regulatory mechanism of CELSR2 expression is still an unsolved mystery, and large-scale HCC genomics research and subsequent functional studies are needed.

## Supplementary information

**Additional file 1: Table S1.** Clinicopathological variables of 74 HCC patients with complete data.

**Additional file 2: Figure S1.** Establishment of knockdown cell models (HepG2 and Hep3B). The effect of CELSR2 knockdown with si-RNAs was verified by western blotting 48 h after transfection.

**Additional file 3: Figure S2.** Full length blots/gels of CELSR2 and GAPDH in Fig. S1 and Fig. 7A are presented.

**Additional file 4: Figure S3.** CELSR2 protein subcellular localization in A-431 and U-251 MG cell lines staining with HPA013952 antibody.

**Additional file 5: Table S2.** Data of mutation diagram of CELSR2 in HCC.

**Additional file 6: Table S3.** Neighboring genes that were significantly associated with CELSR2 mutation.

**Additional file 7: Table S4.** g:Profiler analysis for CELSR2 and 50 frequently altered neighboring genes.

**Additional file 8: Table S5.** The significant gene sets positively and negatively correlated with CELSR2 in LinkedOmics database.

## Data Availability

The datasets used and analysed during the current study are available from the corresponding author on reasonable request.

## Abbreviations

HCC: Hepatocellular carcinoma
OS: Overall survival
UI: Ultrasound imaging
CT: Computer tomography
MRI: Magnetic resonance imaging
AFP: Alpha-fetoprotein
GPC3: Glypican-3
CELSR2: Cadherin EGF LAG seven-pass G-type receptor 2
ING4: Inhibitor of growth 4
TCGA: The cancer genome atlas
GEO: Geneexpression omnibus
GTEx: Genotype-tissue expression
CI: Confidence interval
FDR: False discovery rate
GSEA: Gene set enrichment analysis
CDK2: Cyclin-dependent kinase 2
MAP K1: Mitogen-activated protein kinase 1
ATM: Ataxia-telangiectasia, mutated
RFA: Radiofrequency ablation
RFS: Recurrence-free survival
HPA: Human protein atlas
GO: Gene ontology
CC: Cellular component
BP: Biological process
MF: Molecular function
DMEM: Dulbecco’s modified eagle medium
FBS: Fetal bovine serum
GAPDH: Glyceraldehyde phosphate dehydrogenase
PBS: Phosphate buffered saline
ECL: Enhanced chemiluminescence
qPCR: Quantitative polymerase chain reaction
siRNA: Small inference RNA
