# Supplementary material for: Identification of CELSR2 as a novel prognostic biomarker for hepatocellular carcinoma
Source: BMC Cancer. 2020 Apr 15;20:313. doi: 10.1186/s12885-020-06813-5 (PMC7161135; doi:10.1186/s12885-020-06813-5)
Supplement: Supplementary file 1 — Additional file 1: Table S1. Clinicopathological variables of 74 HCC patients with complete data. [file 12885_2020_6813_MOESM1_ESM.docx]

**Table S1** Clinicopathological variables of 74 HCC patients with complete data

| Variables | HCC patients (N=74) |
| --- | --- |
| Gender (male/female) | 63/11 |
| Age | 47.78±11.14 |
| Tumor size (cm) | 5.09±2.75 |
| Tumor number (single/multiple) | 61/13 |
| Vascular invasion (yes/no) | 24/50 |
| PLT | 179.7±59.39 |
| ALT | 44.07±44.37 |
| AFP | 1129±4600 |
| HbsAg (positive/negative) | 69/5 |
| HBV-DNA | 1.11e6±3.83e6 |
| Operative time (min) | 204±70.38 |
| Liver cirrhosis (yes/no) | 50/24 |
| Tumor differentiation (high/moddle/low) | 12/52/10 |
| MVI (yes/no) | 7/67 |
| CELSR2 (high/low) | 27/47 |
