# Supplementary figures and images for: Identification of CELSR2 as a novel prognostic biomarker for hepatocellular carcinoma
Source: BMC Cancer. 2020 Apr 15;20:313. doi: 10.1186/s12885-020-06813-5 (PMC7161135; doi:10.1186/s12885-020-06813-5)

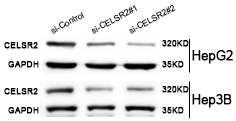

Supplement: Supplementary file 2 — Additional file 2: Figure S1. Establishment of knockdown cell models (HepG2 and Hep3B). The effect of CELSR2 knockdown with si-RNAs was verified by western blotting 48 h after transfection. [file 12885_2020_6813_MOESM2_ESM.tif]

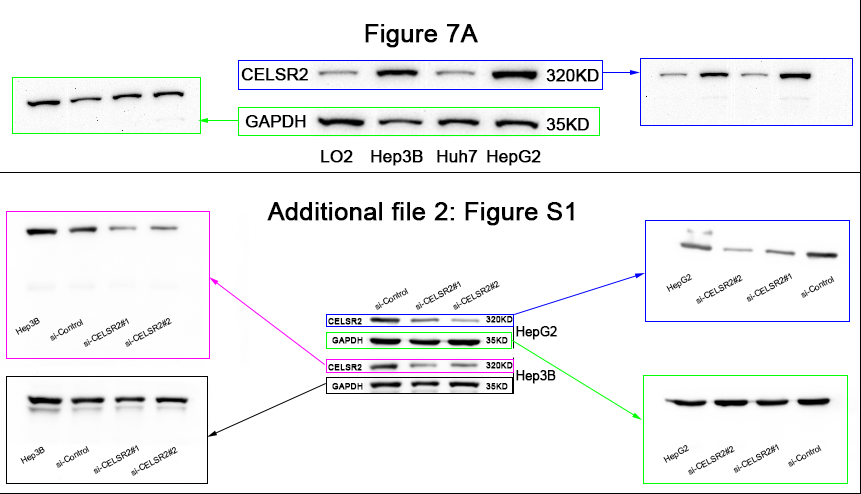

Supplement: Supplementary file 3 — Additional file 3: Figure S2. Full length blots/gels of CELSR2 and GAPDH in Fig. S1 and Fig. 7A are presented. [file 12885_2020_6813_MOESM3_ESM.tif]

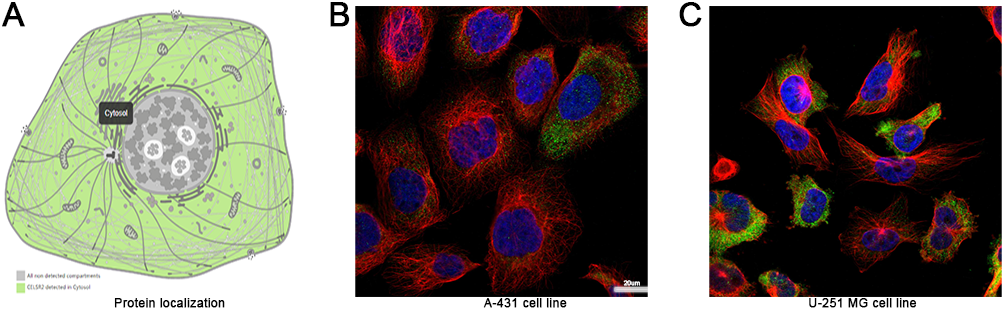

Supplement: Supplementary file 4 — Additional file 4: Figure S3. CELSR2 protein subcellular localization in A-431 and U-251 MG cell lines staining with HPA013952 antibody. [file 12885_2020_6813_MOESM4_ESM.tif]
